# Corticomotor control of the genioglossus in awake OSAS patients: a transcranial magnetic stimulation study

**DOI:** 10.1186/1465-9921-10-74

**Published:** 2009-08-13

**Authors:** Frédéric Sériès, Wei Wang, Thomas Similowski

**Affiliations:** 1 Centre de recherche, Hôpital Laval, Institut universitaire de cardiologie et de pneumologie de l'Université Laval, Quebec City, Quebec, Canada; 2 UPMC Université Paris 6 Pierre et Marie Curie, EA 2397, Paris, France; 3 The 1st Affiliated Hospital of China Medical University, Shen Yang City, Liao Ning Province, PR China; 4 Assistance Publique – Hôpitaux de Paris, Groupe Hospitalier Pitié-Salpêtrière, Service de Pneumologie et Réanimation, Paris, France

## Abstract

**Background:**

Upper airway collapse does not occur during wake in obstructive sleep apnea patients. This points to wake-related compensatory mechanisms, and possibly to a modified corticomotor control of upper airway dilator muscles. The objectives of the study were to characterize the responsiveness of the genioglossus to transcranial magnetic stimulation during respiratory and non-respiratory facilitatory maneuvers in obstructive sleep apnea patients, and to compare it to the responsiveness of the diaphragm, with reference to normal controls.

**Methods:**

Motor evoked potentials of the genioglossus and of the diaphragm, with the corresponding motor thresholds, were recorded in response to transcranial magnetic stimulation applied during expiration, inspiration and during maximal tongue protraction in 13 sleep apnea patients and 8 normal controls.

**Main Results:**

In the sleep apnea patients: 1) combined genioglossus and diaphragm responses occurred more frequently than in controls (*P *< 0.0001); 2) the amplitude of the genioglossus response increased during inspiratory maneuvers (not observed in controls); 3) the latency of the genioglossus response decreased during tongue protraction (not observed in controls). A significant negative correlation was found between the latency of the genioglossus response and the apnea-hypopnea index; 4) the difference in diaphragm and genioglossus cortico-motor responses during tongue protraction and inspiratory loading differed between sleep apnea and controls.

**Conclusion:**

Sleep apnea patients and control subjects differ in the response pattern of the genioglossus and of the diaphragm to facilitatory maneuvers, some of the differences being related to the frequency of sleep-related events.

## Background

The obstructive sleep apnea syndrome (OSAS) is characterized by repetitive episodes of upper airway collapse during sleep that relate to alteration in upper airway stability. Several factors contribute to this upper airway instability (anatomical features, muscular dysfunction, and impaired neuromuscular activation mechanisms) [1-6] but remarkably, upper airway collapse does not occur in awake OSAS patients. This points to wake-related neuromuscular compensatory mechanisms, and hence, possibly, to OSAS-specific changes in the cortical motor control of the upper airway dilators. Indeed, these muscles not only obey brainstem automatic respiratory commands but also in behavioral and voluntary commands, suprapontine in origin, that involve their somatotopic representation within the primary motor cortex. This accounts for the execution of voluntary maneuvers that can be respiratory (e.g. volitional inspiration) but most often are not (e.g. tongue protraction). Both the bulbar and the cortical commands to the upper airway muscles converge to "peripheral" motoneurones where they are integrated and modulate one another [7], as it is the case at the spinal level for phrenic motoneurones [8-10]. This cross-modulation can be studied through the analysis of the electromyographic responses to transcranial magnetic stimulation (TMS) and of the effects of voluntary and involuntary muscle activations on these responses. With this approach, the intensity of the automatic drive to breathe has been shown to increase the excitability of the corticospinal pathway to the diaphragm – facilitation phenomenon – [11,12]. We have demonstrated, in normal individuals, that the response of the genioglossus to TMS is differently influenced by respiratory and non-respiratory maneuvers [7], with a pattern of change that is distinct from that of the diaphragm [7].

From the fact that upper airway collapse does not occur during wake in OSAS patients, that cortical arousal is often required to increase UA muscles activity in OSAS [13] and also that the net activity of the upper airway dilators is higher in awake OSAS patients than in normal individuals [14,15], we hypothesized that the OSAS should be associated with plastic neural changes modifying the interaction between the bulbar and cortical inputs to the upper airway dilators. Considering that the pre-inspiratory activation of UA muscles is a physiological determinant of UA patency [6], we further hypothesized that respiratory and non respiratory-maneuvers would result in specific changes in the pattern of response of UA and respiratory muscles to TMS. To test this hypothesis, we compared the influences of tidal inspiration, resistive loaded breathing and voluntary tongue protraction on the responses of the genioglossus and of the diaphragm to TMS in OSAS patients and normal individuals.

## Methods

### Subjects

Eight healthy male volunteers and thirteen men with untreated OSAS participated in this study. All efforts were made to recruit normal subjects whose age and body mass index were close to those of OSAS. A conventional in-lab full night polysomnographic study (Sandman 4.1, Nellcor Puritan Bennett Ltd., Canada) was completed in every subject. The Laval hospital internal review board approved this protocol and written informed consent was obtained from each subject.

### Measurements

#### EMG

Surface recordings of the right and left costal diaphragmatic EMG activities were obtained with silver cup electrodes placed on the mid-clavicular line in the seventh to eighth right and left intercostal spaces, in such a way as to minimize signal contamination from other muscles [16]. Signal impedence was always lower than 2 kOhm. The good quality of the diaphragmatic EMG signal was assessed by the rise in its raw EMG activity during tidal inspiration. The EMG activity of the genioglossus was recorded by intra-oral electrodes mounted on each side of a mouthpiece made from dental impression [17]. A surface EMG of the dominant-sided abductor pollicis-brevis (APB) was simultaneously recorded with silver cup electrodes as a non-respiratory control muscle. All EMG signals were digitally recorded at a 10 000 Hz sample rate (Digidata 1320, Axon Instrument, Foster City, CA), filtered (10 Hz to 1 KHz) and amplified (Grass CP122, Grass Instrument Co., U.S.A). The EMG activity of the genioglossus during tidal breathing and volitional maneuvers (see below) was also rectified and integrated with a moving averager (MA 1000, CWE, Ardmore, PA) using a time constant of 100 ms. Swallowing, maximal protraction of the tongue over the alveolar ridge and a Müeller maneuver were completed to determine the maximal voluntary activity of the genioglossus [14].

#### Flow

A plastic nasal stent was placed in the anterior nares to prevent nasal collapse. An airtight nasal continuous-positive-pressure mask was then placed over the nose. Instantaneous flow was obtained from a pneumotachograph (Hans Rudolph, model 112467-3850A, Kansas City, MO) connected to the mask. A unidirectional three-way valve could be connected to the pneumotachograph to apply an inspiratory resistance (see below). Flow was digitally recorded at a 2000 Hz sample rate (Digidata 1320, Axon Instrument, Foster City, CA). Subjects were studied lying in a comfortable armchair with a 60 degree inclination and with the head supported by a premolded firm pillow to keep head and neck in the same position during the whole experiment.

### Study Design

All measurements were made with subjects breathing exclusively by the nose. TMS was administered with a Magstim 200 stimulator (Magstim, Whiteland, Dyfed, UK) equipped with a 90 mm circular coil. For each stimulation site, the position of the coil was kept constant by gripping the coil handle to a high precision multi-positional support consisting of two articulated arms (MAN 143, Manfrotto Trading, Milano, Italy). The response of the diaphragm to TMS was assessed with the coil placed at or near Cz (2 cm anterior or 1 cm posterior to Cz), so as to maximize the amplitude of the diaphragmatic motor evoked potential (MEPdi) [18]. The response of the genioglossus to TMS was assessed with the coil placed on the dominant side, over an antero-lateral region (AL) 0 to 6 cm anterior and 6 to 10 cm lateral to the vertex. This area was divided in a 2*2 cm grid using an EEG skin China marker and the coil was successively centered on each intersection point to identify the site yielding the greatest genioglossus motor evoked potential (MEPgg) [19]. Cz-TMS and AL-TMS were delivered in random order.

At each stimulation site, magnetic stimulations were applied in random order in four different conditions (the stimulator being triggered by a timer driven by the zero crossing of the flow signal):

- 0.5 s after the onset of expiration (Exp);

- 0.5 s after the onset of expiration and maximal tongue protraction against the maxillar ridge (Exp+P);

- 1 s after the onset of inspiration (Insp);

- 1 s after the onset of an inspiration loaded by a 100 cm H_2_O/l/s resistance (Insp+R).

Five stimuli were delivered at each stimulation site and in each condition, with the stimulation intensity set at the maximum available output. The intensity was then decreased in a stepwise manner to identify the diaphragm and genioglossus motor thresholds (lowest stimulator output eliciting a 50 μV response on at least 3/5 stimulations).

### Data and Statistical Analysis

All EMG and flow tracings were recorded on a microcomputer (AxoScope software 9.0, Axon Instruments, Inc., USA). The responses of the diaphragm and of the genioglossus to TMS were described in terms of the corresponding motor evoked potentials (MEPdi and MEPgg, respectively). Each single twitch was analyzed separately and results obtained in each given site and condition were pooled in the analysis. The MEP latency was defined as the time elapsed from the stimulus to the first deflection lasting more than 5 ms from baseline. The MEP amplitude was measured from peak to peak. After controlling for univariate normality using the Shapiro-Wilk test, the data were expressed as mean ± SD. The instantaneous flow and integrated GG activity values at which TMS were applied were compared between groups and between maneuvers using a one-way analysis of variance. The effects of the maneuvers on MEP latencies, amplitudes, motor thresholds and genioglossus/diaphragm response patterns to TMS were analyzed using a mixed model analysis, with the subjects as a nested random factor and the interaction terms between the maneuver and the stimulation site as fixed factors. Before this analysis, the equal variances assumption had been verified using the Brown and Forsythe's variation of Levene's statistical test. Multivariate normality had previously verified using Mardia's test. The post-hoc comparisons to assess the effects of the different maneuvers on the muscle responses within groups and within muscles were performed using orthogonal contrasts. These procedures were also completed after adjustment for age and BMI. The relationships between MEP characteristics and the apnea-hypopnea index were studied using linear correlation analysis. Differences were considered significant when p-values ≤ 0.05. All analyses were conducted using the statistical package SAS v9.1.3 (SAS Institute Inc, Cary, NC, U.S.A.).

## Results

The age, body mass index (BMI), apnea-hypopnea index (AHI), neck circumference, instantaneous flow and the baseline GG activities preceding TMS in the different conditions are presented in Table 1. There was no difference between the two groups, except for the AHI.

**Table 1:** Demographic characteristics and genioglossus baseline activity of normal subjects and OSAS patients

	OSAS (n = 13)	Normal (n = 8)
Age (years)	49 ± 6 (40 – 59)	49 ± 5 (40 – 56)

BMI (Kg/m^2^)	31.1 ± 4.2 (25.0 – 39.4)	31.6 ± 4.3 (26.0 – 38.0)

Neck circumference (cm)	41.4 ± 1.9 (38 – 44)	40.0 ± 3.3 (37 – 43)

AHI (n/h)	36.0 ± 15.8*	6.6 ± 3.0

PreTMS Exp GG activity (%max)	8.0 ± 8.9	11.1 ± 6.3

PreTMS Insp GG activity (%max)	8.0 ± 8.9	8.5 ± 8.5

PreTMS Insp+R GG activity (%max)	11.3 ± 9.1	10.3 ± 6.7

PreTMS Exp+P GG activity (%max)	54.1 ± 14.2	49.8 ± 12.7

PreTMS Exp flow (ml/s)	- 513 ± 43	- 494 ± 39

PreTMS Insp flow (ml/s)	490 ± 50	499 ± 80

PreTMS Insp+R flow (ml/s)	179 ± 25	190 ± 23

PreTMS Exp+P flow (ml/s)	- 554 ± 53	- 547 ± 58

Data are presented as Mean ± SD as well as range for anthropometric variables. *: P < 0.01 between normal and OSAS patients.

### MEPs characteristics in OSAS patients and normal controls

**Figure 1:**
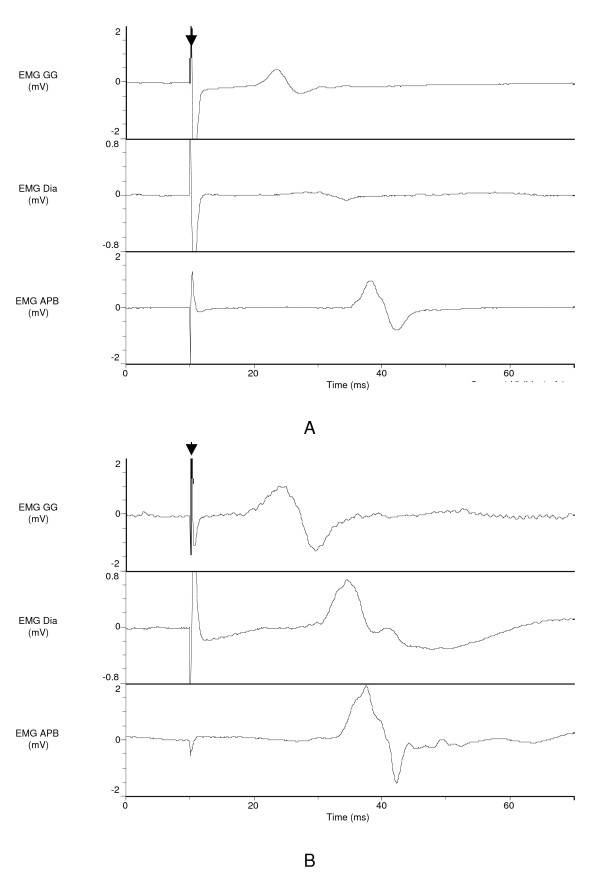
**Representative responses of the genioglossus (GG, top), the diaphragm (Dia, middle) and the abductor pollicis brevis (APB, bottom) to transcranial magnetic stimulation applied anterolaterally during expiration in one normal subject (A) and one patient with OSAS (B)**. The arrow indicates the time of stimulation.

Figure 1 gives representative examples of the responses of the diaphragm, genioglossus and abductor pollicis brevis to AL-TMS during the Exp condition in one OSAS patient and one normal subject.

#### Responses occurrences

AL-TMS and Cz-TMS systematically evoked abductor pollicis brevis responses (Table 2). A diaphragm response, when present, was systematically associated with a genioglossus response, both with AL-TMS and Cz-TMS and in both the OSAS patients and in the normal individuals. Genioglossus responses not associated with a concomitant diaphragm responses occasionally occurred (Table 2). The occurrence of combined GG and diaphragm responses to AL-TMS was significantly more frequent in patients than in controls (P = 0.007) (Table 2).

**Table 2:** TMS-induced EMG responses to 100% TMS intensity at the two TMS sites (% of TMS applied)

	Cz		AL	
	
	normal	OSAS	normal	OSAS
Dia alone	0	0	0	0
GG alone	15.6	13.5	37.5	11.5
Dia +GG	84.4	86.5	62.5	88.5
No response	0	0	0	0

*P *values	0.7		0.007	

The distribution pattern of the diaphragm and genioglossus responses significantly differed between the two groups in AL.

#### Latencies

In the normal subjects, MEPdi and MEPgg latencies were not influenced by the condition underlying AL-TMS and Cz-TMS (Table S1, Additional file 1), but tongue protraction was associated with shortened MEPabp latencies in response to AL-TMS.

**Figure 2:**
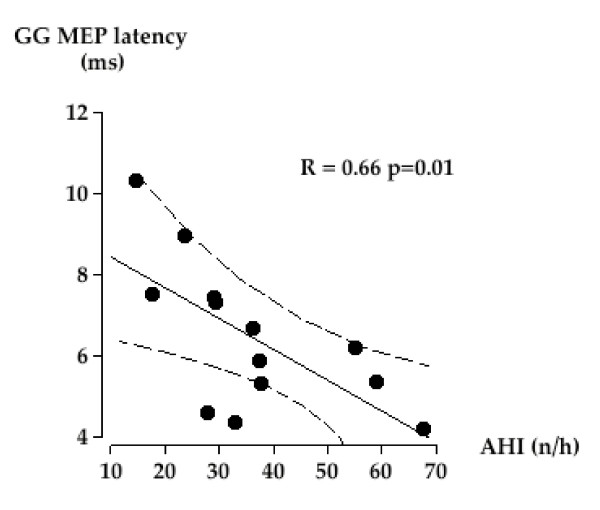
**Relationship between MEPgg latencies and the apnea hypopnea index in the OSAS patients**. Dotted lines represent the 95% confident interval.

In contrast, in the OSAS patients, tongue protraction significantly decreased MEPgg latencies in response to Cz-TMS, and decreased MEPdi latencies in response to AL-TMS (Table S1, Additional file 1). For AL-TMS, the difference in MEPdi and MEPgg latencies in response to Exp + P twitches (normalized for Exp values) significantly differed between OSAS and control subjects (-9.3 ± 2.5% Exp and 6.6 ± 3.1% Exp respectively, p = 0.03). The difference in MEPdi and MEPgg latencies in response to Insp AL-TMS between OSAS and control subjects after adjustment for BMI and age approached significance (12.5 ± 1.0 ms and 9.1 ± 1.4 ms respectively, p = 0.07). MEPapb latencies in response to Cz-TMS were systematically shorter than their "Exp" values in all the conditions. MEPgg latencies in response to AL-TMS were negatively correlated with the apnea-hypopnea index in all conditions, for the anterolateral site of stimulation (Figure 2).

#### Amplitudes

Tidal inspiration and resistive inspiration significantly increased the amplitudes of the MEPgg in response to AL-TMS and Cz-TMS in the OSAS patients, but not in the normal individuals. In both groups the amplitudes of the MEPgg in response to AL-TMS and Cz-TMS increased during tongue protraction in comparison with every other conditions (Table S2, Additional file 2). The amplitudes of the MEPdi responses to AL-TMS and Cz-TMS were similar and not significantly influenced by the underlying condition, both in the OSAS patients and in the normal subjects. However, the difference in MEPgg and MEPdi amplitudes (normalized for Exp values) in response to Cz-TMS applied during Exp + P was significantly higher in control than in SAS (1620 ± 330% Exp and 181 ± 258% Exp respectively, p = 0.02).

The amplitudes of the MEPapb responses to AL-TMS and Cz-TMS were increased by tongue protraction in the OSAS subjects. This phenomenon was observed only for AL-TMS in the normal individuals. The pattern of changes in MEPdia and MEPgg amplitudes to AL-TMS was significantly different between the "respiratory" and the "non-respiratory" conditions in the normal individuals, which was not the case for MEPapb.

### Motor thresholds

**Figure 3:**
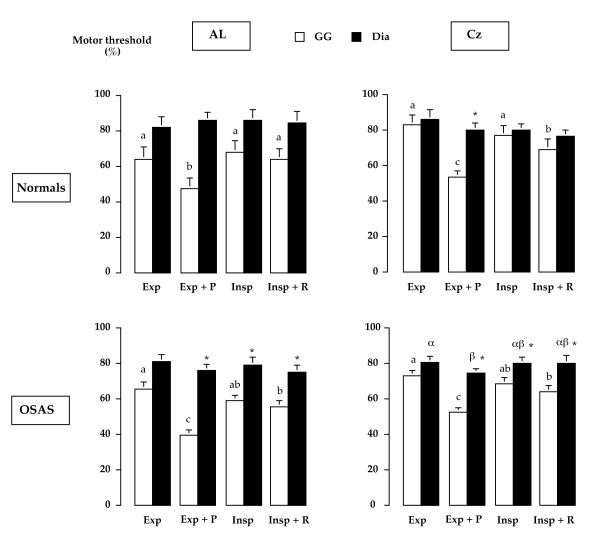
**Diaphragmatic and genioglossus motor thresholds (% maximal stimulation intensity, Mean with indication of 1 SD) in different TMS sites and respiratory conditions**. In each group and for a given muscle and a given stimulation site, columns with different letters are significantly different. * denotes a significant difference between the diaphragm and the genioglossus motor threshold values for a given condition and a given TMS site.

In both groups, the motor threshold of the MEPgg response to AL-TMS and Cz-TMS was significantly lower during tongue protraction than in any other of the conditions studied. The motor threshold of the MEPdi response to Cz-TMS was significantly lower during tongue protraction as compared with the "Exp" condition (74.3 ± 2.4% and 80.4 ± 3.2% respectively) (Figure 3). In the OSAS patients, the MEPgg threshold in response to AL-TMS and Cz-TMS was systematically lower than the MEPdi threshold in any underlying condition (e.g. Cz-TMS during the "Exp" condition 72.9 ± 2.8% vs. 80.4 ± 3.2% respectively) (Figure 3). In the normal individuals, a similar difference existed only in response to Cz-TMS during tongue protraction (Figure 3). The difference between MEPdi and MEPgg thresholds (normalized for Exp values) in response to Cz-TMS applied during Insp + R was significantly higher in SAS than in control (20.9 ± 3.4% Exp and 9.6 ± 3.4% Exp respectively, p = 0.02).

## Discussion

This study shows that the genioglossus/diaphragm response patterns to single shock TMS are different in OSAS patients and in normals. Firstly, coupled responses of the two muscles occur more frequently in OSAS patients (Table 1). Secondly, facilitatory maneuvers have different effects in the two groups (Table 2 and 3), and thirdly these maneuvers differently influence the responsiveness of UA and respiratory muscles to TMS in terms of response latency, amplitude and response threshold. Schematically in OSAS patients as compared to controls, tongue protraction has an enhanced facilitatory effect on the genioglossus response to TMS, inspiratory maneuvers facilitate the response of the genioglossus in terms of latency, amplitude and motor threshold, and tongue protraction cross-facilitates the response of the diaphragm. These differences are observed in spite of similar baseline EMG activities. Of note, the significant correlation that is found between the latencies of the genioglossus responses and the AHI in the OSAS patients, while there is no relationship between the diaphragm latencies and the AHI, reinforces the notion that the genioglossus – diaphragm corticomotor tuning is modified in this population.

### Methodological considerations

The present study was conducted in a male population. Considering the paucity of studies that examined the influence of apnea status on cortico-motor responsiveness, it is not possible to state about the possible influence of gender on the upper airway and diaphragmatic responses to TMS depending on sleep apnea status. Interesting information could come from investigations on the influence of hormonal status (menopause, menstrual cycle) on these responses. It must be pointed out that TMS in this study was performed nonfocally. Our aim was indeed not to separate the genioglossus and the diaphragm responses, but rather to study their coupling during respiratory and non-respiratory maneuvers, in a manner similar to that previously used to study the interactions of the diaphragm bulbospinal and corticospinal commands [9,10,20] or to study some aspects of the motor control of the genioglossus [7,19,21]. Our experimental approach therefore seems adequate to test our hypothesis (OSAS-related changes in the genioglossus and diaphragm motor controls), the non-focal nature of the stimulus not being an obstacle to the interpretation of our results. Of importance, we always positioned the coil in such a way as to obtain optimal EMG responses, and took stringent measures to keep the coil position strictly constant throughout the experiments. The nature of the experimental paradigm and these precautions make us confident about the validity of our observations. Finally, we defined response thresholds in a simplified manner (presence of a response above 50 μV in amplitude in 3 out of 5 attempts, instead of the recommended 5 out of 10 attempts). This choice was mainly motivated by acceptability concerns and was encouraged by previously published studies using a similarly simplified approach [22,23]. The same threshold determination method was used in the two study populations, which should make comparisons possible.

### Increased genioglossus-diaphragm coupling in OSAS patients

The present observations confirm the marked neurophysiological coupling between the genioglossus and the diaphragm that was previously evidenced in normal subjects [7] through the existence of cross-facilitation during respiratory maneuvers. Here we show that this coupling is particularly marked in OSAS patients. This could result from plastic adaptive changes occurring at the level of the UA muscles brainstem motoneurons, their cortical representation, or both.

Our findings are indeed consistent with observations depicting a modified behavior of the UA dilator muscles in OSAS patients. For instance, an OSAS-related increase in the tidal phasic activity of these muscles has been interpreted as compensatory of smaller pharyngeal dimensions and of an exaggerated pharyngeal collapsibility [14,15]. This increase could be due to an augmented gain of the reflex response of the UA dilators to negative upper airway pressure. However, the increased phasic genioglossus activity persists after unloading [24], and the tonic activity of this muscle is also elevated in OSAS patients [15]. This points to factors other than an increased reflex gain, as, for example, an increase in the respiratory-related drive to upper airway dilators. Such an increase would be consistent with the modulation of the activity of hypoglossal motoneurons by inputs from respiratory neurons that has been reported in animal studies [25-27], and also consistent with the pre-activation of UA muscles before the onset of inspiration [28]. It would explain the increased MEPgg amplitude and the decreased MEPgg motor thresholds that we observed during inspiratory TMS in our OSAS patients.

Additional interesting information come from the influence of respiratory and volitional maneuvers on the difference in genioglossus and diaphragm cortico-motor responses between SAOS and controls. Our results are consistent with a predominant rise in diaphragm rather than genioglossus corticomotor responsiveness during active tongue protrusion in OSAS. On the other hand, the facilitatory effect of tidal inspiration on genioglossus pre activation tended to be higher in OSAS than in controls also with a preferential increase in UA muscles excitability during inspiratory loading in sleep apnea patients. Considering the importance of the balance between UA and respiratory muscles cortico-motor acivation process (amount of UA muscles pre-activation and respective activity of these muscles) on the occurrence of UA closure, it will be particularly interesting to investigate the influence of sleep on this motor activation pattern.

### Putative sites of the excitability changes

Facilitatory maneuvers modify TMS responses by increasing synaptic excitability at either the motor cortex and/or the spinal motoneurons [29] (or, in the case of upper airway muscles, the "peripheral" bulbar motoneurons). Single shock TMS as used in our study is not sufficient to discriminate between cortical and "peripheral" facilitation. Other TMS variables such as the central silent period or intracortical excitability studied with paired stimulations are necessary to do so. Our study was designed to test the hypothesis that patients suffering from the obstructive sleep apnea syndrome exhibit changes in the corticomotor control of the upper airway dilator muscles. In the absence of previous data of this kind, we chose a global test of corticomotor function to provide a first answer to the research question while keeping the study feasible and acceptable to the participants. Our results are consistent with a recent report describing, in OSAS patients, increased genioglossus single motor unit action potential and modifications in timing and firing inspiratory frequencies [30]. Further work will be needed to understand the mechanisms underlying our observations.

### Specificity of theGG corticomotor activation profile in OSAS

We found that, in OSAS patients studied awake, the MEPgg latency was inversely correlated with the AHI. In other words, the more severe was the OSAS and hence the instability of the upper airway, the more responsive was the genioglossus to TMS and hence, possibly, the higher the drive to the genioglossus. It is therefore tempting to hypothesize that the TMS results unveil a compensatory mechanism that prevents upper airway collapse during wakefulness. A relationship between the AHI and MEPgg latency was only observed in the OSAS group, supporting the reality of OSAS-related changes in the corticomotor control of the genioglossus.

In this view, it is of importance to note that in the OSAS group the influence of the respiratory maneuvers on the MEP characteristics and on the motor threshold was marked for the genioglossus but lacked for the diaphragm. This complements previous studies having shown that the TMS responsiveness of limb muscles is unaltered in OSAS patients [31]. In our patients, the genioglossus motor threshold was lower than the diaphragm motor threshold in all the study conditions in the OSAS group, but this pattern was only observed for Cz-TMS in response to a specific, non respiratory, genioglossus activation in the normal group (Exp+P condition, where TMS was delivered at end expiration during voluntary tongue protraction). This discrepancy between the genioglossus cortico-motor responsiveness and the diaphragm one can be put in parallel with previous observations. Indeed, it has been shown that in response to a progressive isocapnic hypoxic challenge, the activity of the genioglossus increases in a steeper manner than that of the diaphragm in OSAS patients as compared to controls [32]. This has been seen as a protective mechanism for the maintenance of upper airway patency in situations promoting their instability.

### Baseline genioglossus activity

We found a similar baseline genioglossus activity in our OSAS patients and our control subjects, in contrast with previous observations [14,15] but in line with others [33]. This difference between our study and others could result from differences in the EMG recording technique or in the sharpness of the between-groups weight matching. In this regard, our OSAS and control subjects were remarkably similar except for the AHI. The differences that we observed in terms of TMS responses are thus not likely to be explained by age or anthropometric differences, or even by differences in baseline EMG activity. This illustrates the usefulness of TMS to assess the central neuromuscular drive to UA dilator muscles particularly when conventional EMG recordings are not contributive.

## Conclusion

This study provides a strong clue to a modification in the corticomotor control profile of certain upper airway muscles in OSAS during wakefulness. Further experiments are needed to evaluate the responsiveness of upper airway dilators other than the genioglossus and particularly to compare phasic and tonic muscles. The possibility to apply TMS during sleep [10,31] also opens the way to a direct exploration of the influence of sleep on upper airway and inspiratory neuromuscular activation processes. This approach may also prove very useful to evaluate pharmaceutical treatments of the OSAS aimed at modulating the activity of UA dilator during sleep.

## Abbreviations

APB: Abductor pollicis brevis; AL: antero-lateral region; BMI: body mass index; MEPdi: diaphragmatic motor evoked potential; Exp: expiration; MEPgg: genioglossus motor evoked potential; Insp: inspiration; Insp+R: inspiration loaded; OSAS: obstructive sleep apnea syndrome; MEP: motor evoked potential; Exp+P: tongue protraction; TMS: transcranial magnetic stimulation; UA: upper airway.

## Competing interests

The authors declare that they have no competing interests.

## Authors' contributions

FS conceived of the study, elaborated its design and contributed to its coordination. TS and WW participated in the revision of the design of the study. WW participated to data collection and interpretation. All authors participated in and helped to draft the manuscript. All authors read and approved the final manuscript.

## Supplementary Material

Additional file 1 **Table S1**. Mean ± SD values (ms) of GG, Dia and APB MEP latencies in response to TMS applied in different sites and respiratory conditions. In each group and for a given muscle and a given stimulation site, rows connected by red bars are significantly different. Click here for file

Additional file 2 **Table S2**. Mean ± SD values (mV) of GG, Dia and APB MEP amplitudes in response to TMS applied in different sites and respiratory conditions. In each group and for a given muscle and a given stimulation site, rows connected by red bars are significantly different. Click here for file
