# Supplementary material for: Corticomotor control of the genioglossus in awake OSAS patients: a transcranial magnetic stimulation study
Source: Respir Res. 2009 Aug 13;10(1):74. doi: 10.1186/1465-9921-10-74 (PMC2738672; doi:10.1186/1465-9921-10-74)
Supplement: Additional file 1 — Table S1. Mean ± SD values (ms) of GG, Dia and APB MEP latencies in response to TMS applied in different sites and respiratory conditions. In each group and for a given muscle and a given stimulation site, rows connected by red bars are significantly different. [file 1465-9921-10-74-S1.pdf]

Table S1. Mean ± SD values (ms) of GG, Dia and APB MEP latencies in response to TMS applied in different sites and respiratory conditions. In each group and for a given muscle and a given stimulation site, rows connected by red bars are significantly different.

		AL			Cz		
		GG	Dia	APB	GG	Dia	APB
Normals	Exp	7.11±1.54	17.03±0.83	24.21±0.80	9.80±0.89	17.65±0.66	24.62±0.62
	Exp+P	6.95±1.51	17.35±0.46	23.24±0.69	8.94±0.95	17.32±0.56	24.74±0.68
	Insp	7.43±1.64	17.23±0.92	24.05±0.70	9.57±0.92	16.32±0.67	24.43±0.54
	Insp+R	7.15±1.52	17.62±0.80	24.01±0.56	9.08±0.82	17.26±0.88	24.52±0.62
OSAS	Exp	6.57±0.73	18.50±0.49	23.83±0.43	10.76±0.48	18.73±0.54	24.45±0.46
	Exp+P	6.46±0.51	17.70±0.72	23.41±0.41	8.84±0.62	18.00±0.49	23.33±0.47
	Insp	6.35±0.69	18.42±0.44	23.51±0.36	10.15±0.54	18.56±0.53	23.84±0.43
	Insp+R	6.51±0.73	18.70±0.75	27.48±0.54	9.80±0.51	18.21±0.48	23.85±0.46
