# Supplementary material for: Corticomotor control of the genioglossus in awake OSAS patients: a transcranial magnetic stimulation study
Source: Respir Res. 2009 Aug 13;10(1):74. doi: 10.1186/1465-9921-10-74 (PMC2738672; doi:10.1186/1465-9921-10-74)
Supplement: Additional file 2 — Table S2. Mean ± SD values (mV) of GG, Dia and APB MEP amplitudes in response to TMS applied in different sites and respiratory conditions. In each group and for a given muscle and a given stimulation site, rows connected by red bars are significantly different. [file 1465-9921-10-74-S2.pdf]

Table 4. Mean ± SD values (mV) of GG, Dia and APB MEP amplitudes in response to TMS applied in different sites and respiratory conditions. In each group and for a given muscle and a given stimulation site, rows connected by red bars are significantly different.

		AL			Cz		
		GG	Dia	APB	GG	Dia	APB
Normals	Exp	0.81±0.32	0.33±0.25	2.69±0.62	0.62±0.25	0.22±0.10	2.63±0.78
	Exp+P	1.26±0.37	0.22±0.08	3.80±0.45	1.35±0.37	0.31±0.07	2.87±0.69
	Insp	0.76±0.29	0.38±0.26	2.98±0.52	0.73±0.32	0.26±0.09	2.56±0.82
	Insp+R	0.78±0.28	0.47±0.26	3.23±0.66	0.94±0.31	0.34±0.13	2.89±0.80
OSAS	Exp	0.61±0.12	0.59±0.19	2.35±0.52	0.67±0.17	0.36±0.09	2.31±0.42
	Exp+P	1.29±0.17	0.78±0.27	3.19±0.52	1.46±0.18	0.63±0.18	3.47±0.55
	Insp	0.76±0.19	0.63±0.20	2.68±0.56	0.80±0.20	0.44±0.16	2.38±0.42
	Insp+R	0.79±0.14	0.78±0.35	3.13±0.62	0.98±0.23	0.54±0.19	2.86±0.58
